# Smokeless Tobacco Use: A Risk Factor for Hyperhomocysteinemia in a Pakistani Population

**DOI:** 10.1371/journal.pone.0083826

**Published:** 2013-12-23

**Authors:** Mohammad Perwaiz Iqbal, Mohsin Yakub

**Affiliations:** Department of Biological and Biomedical Sciences, Aga Khan University, Karachi, Pakistan; University of Louisville, United States of America

## Abstract

**Background:**

Smokeless tobacco (ST) use is highly prevalent in the South Asian populations. While there have been a number of reports on association of ST consumption with cancer, very few studies have been conducted to investigate its relationship with cardiovascular disease. Hyperhomocysteinemia is a well-recognized risk factor for cardiovascular disease; however, its association with ST use has never been investigated. The objective of this study was to evaluate the relationship of ST use with hyperhomocysteinemia in an urban Pakistani population.

**Methodology/Principal Findings:**

In a cross-sectional study for assessment of risks of hyperhomocysteinemia, 872 healthy adults (355 males and 517 females of age range 18–60 years) were recruited from a low-income population in Karachi, Pakistan. A detailed questionnaire was administered which included information about smoking, non-smoking, use of ST alone (chewing as well as sniffing) and use of ST with betel nuts. Fasting serum/plasma levels of homocysteine, folate, vitamin B12 and pyridoxal phosphate (PLP; a coenzymic form of vitamin B6) were analyzed. In this population, 43.4% males and 15.5% females were found to be regular users of ST products. Laborers and vendors were the major ST consumers. Smoking was not found to be associated with plasma/serum concentrations of homocysteine, folate, vitamin B12 and PLP. However, homocysteine concentrations in the group which consumed ST alone and the group which consumed ST along with betel nut were significantly higher compared to the non-user group (17.7±7.5 µmol/L, 25.48 µmol/L vs. 11.95 µmol/L, respectively; p<0.01). Odds ratio for the association of hyperhomocysteinemia (>15 µmol/L) was 11-fold higher in the ST-consumer group compared to the non-user group, [OR (95%CI)  = 11.34 (7.58–16.96); p<0.001], when the model was adjusted for age, gender, folate and vitamin B12 status.

**Conclusion:**

This study shows a positive association between ST consumption and hyperhomocysteinemia in a low-income urban Pakistani population.

## Introduction

There is an epidemic of smokeless tobacco (ST) use in South Asia [1]–[3]. Percentages of ST consumption among adults in India, Sri Lanka and Myanmar are 20%, 15.8% and 20.8%, respectively [4]–[6], while in Bangladeshi women ST consumption has been reported to be 27.9% [2]. In Pakistan, a few studies that have been carried out indicate its prevalence in young adults to be 16.1% to 20% [7], [8]. It is ironic that the Pakistani public at large perceives tobacco chewing to be a harmless activity; while it has been shown that its long-term use can lead to serious health problems because ST products have been shown to contain 30 cancer causing substances, in addition to nicotine which causes addiction [9]. ST used in Pakistan is mainly in two forms; ST chewing/sniffing, for example, *naswar* - a product made from fresh tobacco leaves, calcium oxide and wood ash [10] and ST chewing with betel nut. Betel nut or areca nut is the seed of the *Areca catechu* tree and is most commonly used with ST in South Asia. It is used in the form of *betel quid* - betel leaf with or without tobacco, areca nut and slaked lime, or as *Gutka* - a combination of powdered tobacco with betel nut and slaked lime [11]. While a number of studies have been carried out on the association of ST consumption with cancer, there have been very few reports on its relationship with cardiovascular disease. According to the INTERHEART Study carried out in 52 countries, individuals who only chewed tobacco had a significantly increased risk [Odds ratio 2.23] of first myocardial infarction compared to those who never used tobacco [12]. Similarly, a recent study carried out in India showed that chewing even a slight amount of tobacco could cause narrowing of major heart arteries by over 14% [13]. These reports indicate a strong relationship between use of ST and coronary heart disease (CHD). However, the mechanism by which it increases the risk of CHD is unclear.

Hyperhomocysteinemia (> 15 µmol/L) is an established risk factor for cardiovascular disease [14], [15]. However, its association with ST use has never been reported. The objective of the current study was to investigate a possible relationship between use of ST and hyperhomocysteinemia in a low income urban locality in Karachi.

## Methods

### Ethics Statement

This study protocol was approved by the Ethics Review Committee of the Aga Khan University, and prior written informed consent was obtained from all individuals who participated in this study.

### Participants’ Enrollment

In a cross-sectional study for the assessment of risks of hyperhomocysteinemia, 872 healthy adults (age 18–60 years) were recruited from Sultanabad, a low income urban area in the East region of Karachi. The study procedures and primary outcomes have been reported in detail previously [16]–[18]. Briefly, a systemic random sampling was adopted along with detailed questionnaire which gathered demographic characteristics, occupational status, educational status and information about smoking, non-smoking, ST chewing/sniffing and ST chewing with betel nut. Category of ST users included those who had been using ST products habitually, at least 3–4 times per week for the last 6 months, while occasional users and one time users were placed in the category of non-users.

### Blood Sampling and Measurement of Biomarkers

Blood samples were obtained in fasting state. Plasma homocysteine was determined using an immunoassay based kit following manufacturer’s instruction [Abbott’s Laboratories Ltd., Pakistan], while serum folate, vitamin B12 and plasma pyridoxal phosphate [PLP, a co-enzymic form of vitamin B6] were determined using radio assays [19]–[21]. Serum cholesterol was determined using a kit method [Roche Diagnostics, USA].Whole blood lead (Pb) was measured on graphite furnace using Hitachi Z-8000 Atomic Absorption Spectrometer with Zeeman’s background correction [18]. To ensure validity of our measurements, appropriate controls with high and low concentrations of analytes were used for every assay.

### Statistical analysis

All statistical analyses were carried out with the help of Statistical Package for Social Sciences® (SPSS) software version 13 for Windows® Apache Software Foundation, USA.

Continuous variables such as age, homocysteine, folate, vitamin B12, PLP, cholesterol and blood Pb were expressed as means±SD, and categorical variables such as gender, educational status, ethnicity, occupation, smoking, tobacco chewing/sniffing alone and tobacco chewing with betel nut as n (%). Percentages among never users, only ST users and those using ST along with betel nut were compared using Pearson Chi-square. Mean concentrations of biomarkers among smokers and non-smokers were compared using Independent sample t test, while ANOVA followed by Tukey’s HSD test for multiple pair-wise comparisons were used to compare mean concentrations of these biomarkers among non-users, only ST users and those who used ST along with betel nut. Logistic regression was used to evaluate the association of ST use with hyperhomocysteinemia, while adjusting for significant factors from the chi-square analysis.

## Results

Demographic characteristics of ST users and non-users have been shown in Table 1. In a total of 872 participants, 43.4% males were ST users compared to 15.5% females.

**Table 1:** Demographic characteristics of participants who were users and non-users of smokeless tobacco (ST).

		Non-users		ST only		ST plus betel nut	Pearson Chi-
		(*n* = 638)		(*n* = 88)		(*n* = 146)	Square
		*n* (%)		*n* (%)		*n* (%)	
**Gender**							
Male		201		37		117	< 0.001
		(56.6)		(10.4)		(33.0)	
Female		437		51		29	
		(84.5)		(9.9)		(5.6)	
**Ethnic Origin**							
Hindko/Hazara & Punjabis in north of Pakistan			329 (70.4)		59 (12.6)	79 (16.9)	0.039
Pathans			232 (77.1)		24 (8.0)	45 (15.0)	
Others			77 (74.0)		5 (4.8)	22 (21.2)	
**Education status**							
No education		272 (81.7)		33 (9.9)		28 (8.4)	< 0.001
Up to 8^th^ grade		132 (67.7)		19 (9.7)		44 (22.6)	
Up to 10^th^ grade		136 (67.0)		20 (9.9)		47 (23.1)	
College/University		98 (69.5)		16 (11.3)		27 (19.1)	
**Occupation**							
House wives/unemployed		427 (84.4)		49 (9.7)		30 (5.9)	< 0.001
Shopkeepers & office workers		49 (61.3)		10 (12.5)		21 (26.3)	
Laborers & vendors		119 (53.3)		22 (9.9)		82 (36.8)	
Students		43 (68.3)		7 (11.1)		13 (20.6)	

Regarding ethnic origin, 467 (53.6%) belonged to Hazara, Hindko and north Punjab group, while 301 (34.5%) were Pathans who had migrated from Khyber Pakhtunkhwa Province of Pakistan. The rest 104 (11.9%) belonged to other ethnic groups such as Mohajir, Sindhi, and Balochi. Hazara/Hindko speaking participants along with Punjabis in the adjoining areas of north Punjab were the major consumers of ST in this cohort.

*Paan* (fresh betel leaf), *Gutka* and betel nut were more popular among subjects who migrated from Hazara and north Punjab, while *naswar* was preferred ST among Pathans from the Khyber Pakhtunkhwa Province. There was no significant difference in the mean ages of those who never used ST and those who used ST alone or with betel nut (ANOVA p = 0.105).

In terms of occupation of participants, laborers and vendors were the main ST consumers (46.7%) followed by office and factory workers and shopkeepers (38.8%), students (31.7%) and housewives and unemployed persons (15.8%). No significant difference was found in mean body mass index (BMI) values among the non-users, ST users and ST users along with betel nut groups (p = 0.14). Cigarette smoking had no significant effect on plasma/serum concentrations of homocysteine, folate, vitamin B12 and PLP and blood Pb in males in this cohort (Table 2).

**Table 2:** Effect of smoking on concentrations of plasma/serum homocysteine, folate, vitamin B12 and PLP and blood Pb in normal healthy adult males.

Mean±SD			
Variables	Concentration		p-value*
	Nonsmokers	Smokers	
	(*n* = 276)	(*n* = 79)	
**Homocysteine (µmol/L)**	19.80±12.46	19.36±10.0	0.77
**Folate (ng/mL)**	5.67±3.52	5.35±4.0	0.49
**Vitamin B12 (pg/mL)**	431±194	413±186	0.46
**PLP (nmol/L)**	37.6±27.6	34.2±32.9	0.35
**Blood Pb (µg/dL)**	11.5±5.3	12.5±5.8	0.13

* Independent sample t-test was used to compare mean values between nonsmokers and smokers.

Mean levels of plasma homocysteine were significantly increased (p<0.001) in the group which consumed ST along with betel nut compared to the group of non-users (Table 3). Similarly, mean levels of serum folate and vitamin B12 were significantly lower in the ST users group compared to the non-users group (p<0.001 and p = 0.003, respectively).

**Table 3:** Effect of smokeless tobacco (ST) consumption alone or with betel nut on concentrations of plasma/serum homocysteine, folate, vitamin B12, PLP and cholesterol and blood Pb in normal healthy adults.

Variable	Concentration			p-value*
	Smokeless Tobacco Use			
	Never	Alone	With betel nut	
	(*n* = 638)	(*n* = 88)	(*n* = 146)	
**Homocysteine(µmol/L)**	11.95±5.5	17.7±7.5	25.48±15	<0.001
**Folate (ng/mL)**	7.1±4.7	6.07±4.1	4.68±3.1	<0.001
**Vitamin B12 (pg/mL)**	456±241	406±203	391±173	0.003
**PLP (nmol/L)**	34.2±36.1	28±18	31.5±23	0.2
**Cholesterol (mg/dL)**	158±35	164±35	162±38	0.17
**Blood Pb (µg/dL)**	11.4±5.4	12.6±5.8	12.3±5.7	0.05

Mean±SD.

* p-value compares the mean values among the non-users, ST users and ST along with betel nut chewers using one way ANOVA. Tukey’s HSD multiple pair-wise comparison showed that ST users (with and without betel nut) had significantly higher levels of plasma homocysteine (p<0.001) compared to non-users of ST. Regarding serum folate and serum B12 levels, the group chewing tobacco along with betel nut was found to have significantly lower levels of folate and vitamin B12 compared to the non-users group (p<0.001 and p = 0.003, respectively).

In order to evaluate the association of ST use with hyperhomocysteinemia, logistic regression analysis was carried out. As shown in Table 4, the odds ratio (OR) for the association of hyperhomocysteinemia was nearly 15-fold among ST users compared to non-users. When the model was adjusted for age, gender, folate status, vitamin B12 status, blood Pb levels, BMI and serum cholesterol, the OR was still 11.49. Odds for the association of folate deficiency and vitamin B12 deficiency was nearly 2-fold in ST users compared to non-users (Table 5) indicating a relationship between ST use and deficiencies of folate and vitamin B12.

**Table 4:** Association of hyperhomocysteinemia^1^ with smokeless tobacco (ST) use.

	Non-users	ST users alone & with betel nut
	(*n* = 638)	(*n* = 234)
**Crude** 2	1	14.89 (10.38–21.36)*
**Adjusted** 3	1	11.34 (7.58–16.96)*
**Adjusted** 4	1	11.35 (7.58–16.99)*
**Adjusted** 5	1	11.49 (7.66–17.23)*

1 Plasma homocysteine >15 µmol/L.

2 Values are OR (95% CI) from logistic regression, *p<0.001.

3 Values are OR (95% CI) from logistic regression adjusted for age (y), gender, folate status (≤3.5 ng/mL, >3.5 ng/mL) and, vitamin B12 status (≤200 pg/mL, >200 pg/mL), *p<0.001.

4 Values are OR (95% CI) from logistic regression adjusted for age (y), gender, folate status (≤3.5 ng/mL, >3.5 ng/mL), vitamin B12 status (≤200 pg/mL, >200 pg/mL) and blood lead (<10 µg/dL, ≥10 µg/dL), *p<0.001.

5 Values are OR (95% CI) from logistic regression adjusted for age (y), gender, folate status (≤3.5 ng/mL, >3.5 ng/mL), vitamin B12 status (≤200 pg/mL, >200 pg/mL), blood lead (<10 µg/dL, ≥10 µg/dL), BMI and cholesterol. *p<0.001.

**Table 5:** OR for the association of folate and vitamin B12 deficiencies^1^ with smokeless tobacco (ST) use.

	Folate deficiency		Vitamin B12 deficiency	
	Non user	ST users alone and with betel nut	Non user	ST users alone and with betel nut
**Crude^2^**	1	2.32(1.68–3.20)**	1	1.74(1.09–2.78)*
**Adjusted^3^**	2	2.11(1.50–2.96)**	2	1.82(1.11–3.00)*

1. Folate deficiency (serum folate <3.5 ng/mL): Vitamin B12 deficiency (serum vitamin B12<200 pg/mL).

2. Values are OR(95% CI) from logistic regression, *p<0.05, **p<0.001.

3. Values are OR(95% CI) from logistic regression adjusted for age (y) and gender; *p<0.05, **p<0.001.

## Discussion

The high prevalence of ST consumption in Pakistani urban males compared to females (43.4% vs. 15.5%) is consistent with previous studies [22], [23]. The ratio of male to female ST users in this study is very similar to ratios reported in earlier studies from India and Pakistan [4], [22], [23]. Moreover, significantly greater proportions of males chewed tobacco along with betel nut as compared to those who only chewed or sniffed tobacco (*naswar*). All of them were males and ten individuals among them were cigarette smokers as well. Since none of them chewed betel nut, they were included in the group which consumed ST alone.

In this study, those who had no education were less likely to be involved in tobacco chewing or sniffing compared to those who went to school or college. This is suggestive of a relationship between education and ST use. However, use of ST among college and university level educated individuals was not different from those who had school level education up to 8^th^ or 10^th^ grade. These findings are in contrast to the results reported by Pandey et al. who have shown a correlation between ST use and less than 5 years of school education [4]. This indicates that relationship of ST use and education could vary from one population to another. Perhaps, factors such as advertisements in the media and social environment might be playing a role in getting them attracted to ST use.

Recent reports have indicated high proportions of ST consumers in certain occupations [24]. In the present study, laborers and vendors were found to be the main ST consumers. These findings conform well to those reported from India where ST consumption has been found to be more prevalent in the semi-skilled workers and laborers [4]. This also shows that people in certain occupations requiring physical work are more prone to getting addicted to the use of ST.

Cigarette smoking has been reported to be associated with increased levels of plasma homocysteine and decreased levels of serum folate and vitamin B12 [25]. However, cigarette smoking had no significant effect on mean concentrations of plasma homocysteine, serum folate, vitamin B12, PLP and blood Pb in males in the present study. Since there were no female cigarette smokers in this cohort, we confined our analysis to only males. We are mindful of the fact that smoking by females is a taboo in certain population groups in Pakistan. Therefore, the possibility that some of the female smokers preferred not to accept this fact cannot be ruled out. Urine cotinine is a biological marker of tobacco use [25]. However, we did not monitor cotinine levels in urine of participants. This would remain one of the limitations of the study.

The most important finding of this study is significantly increased mean level of homocysteine (p<0.001) in the group which consumed ST along with betel nut. The mean homocysteine concentration (25.48±15 µmol/L) in this group was well above 15 µmol/L- maximum upper limit of the normal level [26], and appears to be due to decreased concentrations of folate and vitamin B12. This is suggestive that ST consumers, especially those who use these products along with betel nut, would be more prone to developing hyperhomocysteinemia. This association of ST use with hyperhomocysteinemia was further confirmed by running a logistic regression model.

The odds of having hyperhomocysteinemia was nearly 15-fold among ST users compared to non-users. When the model was adjusted for age, gender, folate status and vitamin B12 status, the OR was still 11.34. However, further adjustment with blood Pb levels, BMI and serum cholesterol resulted into a marginal increase in OR (11.49). There have been a few reports which indicated increased risk of cardiovascular disease among consumers of ST compared to nonusers. Bolinder et al. [27] in a large Swedish cohort reported a significantly increased risk for mortality from cardiovascular disease in male exclusive users of ST compared to those who never used tobacco (age-adjusted relative risk 1.4, 95%CI, 1.2–1.6). Similarly, Pandey et al. reported an association between ST consumption and hypertension in an adult rural population of India [4].

A more detailed account of the impact of ST products on cardiovascular disease was presented by Piano et al. in the form of a Policy Statement from the American Heart Association [28]. Though impact of ST products on various risk factors for cardiovascular disease such as C reactive protein, total cholesterol, HDL-cholesterol, LDL-cholesterol, triglycerides, fibrinogen etc. was discussed in this paper, it did not touch upon the relationship of ST and homocysteine. Therefore, our study is important and unique in the sense that it reports an association between use of ST and hyperhomocysteinemia.

Significantly decreased serum concentrations of folate and vitamin B12 and increased levels of plasma homocysteine in ST users point towards a relationship between ST consumption and hyperhomocysteinemia in this cohort. This was further supported by the observation that odds for the association of folate deficiency and vitamin B12 deficiency was nearly 2-fold in ST users compared to non-users. We could not find any report in the literature showing an association of ST use and deficiencies of folate and vitamin B12. However, chronic cigarette smoking has been found to be associated with diminished folate and vitamin B12 status [25], [29].

It would also be important to discuss the possible role of some of the components of ST products in causing hyperhomocysteinemia. A report from the Center of Environmental Studies, Pakistan Council of Scientific and Industrial Research indicated high contents of 3 toxic metals, lead, cadmium and nickel in ST products of Pakistan [30]. We have previously reported an association of Pb with plasma homocysteine in a Pakistani population [18]. However, adjustment of regression model with blood Pb in the present study did not increase the odds of hyperhomocysteinemia. This shows that the mechanism by which ST consumers are having higher levels of plasma homocysteine is independent of their blood Pb levels. There have been reports that nickel and arsenic affect homocysteine metabolism [31]. There is a possibility that these metals might be contributing to high levels of homocysteine either by decreasing the activity of enzymes involved in homocysteine catabolism or in some manner affecting the methylation of homocysteine into methionine.

Nitrosamines are the most harmful chemicals present in ST products. Nitrosamines are formed during growing, curing, fermenting and aging of tobacco [32]. The nitrosamine content of ST products exceeds beyond 1000-fold the nitrosamine content allowed by FDA in other food products/drinks [33]. They have been known to cause oxidative stress and cellular injury due to the involvement of free radicals [34]. It is possible that nitrosamines might be influencing the activity of certain enzymes in homocysteine metabolism. In a previous communication, we have shown that betel nut use does not cause increase in plasma homocysteine levels in a rat model [35]. Therefore, we suggest that increased levels of homocysteine among ST users are most likely due to tobacco and not betel nut. We did not have in the questionnaire information about those who only consumed betel nut. Moreover, information was also lacking about the frequency with which ST products were used daily or weekly, the number of years of ST use and reasons for using these products.

In spite of these limitations, the study does show an association between ST consumption and hyperhomocysteinemia in a low income urban population in Karachi. However, more in-depth studies would be required to unravel the molecular mechanism for these increased levels of plasma homocysteine in ST consumers.

There is an epidemic of cardiovascular diseases in South Asia [36], [37]. Men are more susceptible to developing cardiovascular disease compared to women, and one of the possible factors could be increased level of plasma homocysteine in men [16]. The present study shows an association between ST use and hyperhomocysteinemia and significantly higher proportion of male ST users compared to females (vide supra). This is suggestive that Pakistani men, especially those engaged in ST use appear to be more prone to developing hyperhomocysteinemia. High prevalence of ST use among men could be because of the fact that males in this region have more social freedom and easy access to ST products [38] or nature of their work, especially among the labor class, requires them to use ST products habitually. In order to combat the spread of cardiovascular disease in South Asia, it is important that effective community based ST prevention and cessation programs having all the 3 components such as education, information and counseling must be initiated [39]. Moreover, government agencies and law makers should be involved in the control of unregulated spread of ST products.

## Conclusion

In a cross-sectional survey, a positive association was found between ST consumption and hyperhomocysteinemia in a low income urban population in Karachi, Pakistan.
